# Dr. John P. Holmes

**Published:** 1885-12

**Authors:** 


### Obituary.

---

Dr. John P. Holmes.—It is with great regret that we note the death of one, who for many years has been prominently and most favorable known to the dental profession of the South as a gentleman of the highest character and of sterling integrity. Dr. Holmes was born at Spring Ridge, Hinds county, Mississippi, July 18th, 1842.

He died of apoplexy, at his residence in Vineville—suburban village to Macon, Ga., September 2d, 1885, in the forty-third year of his age. He was the son of Mrs. R. L. and Dr. Henry J. Holmes; the latter a prominent physician in Mississippi, who died in 1875.

Dr. Holmes graduated at the Ohio College of Dental Surgery in the class of 1868, and since 1878 has been connected with his brother, Dr. W. R. Holmes, in the dental depot business in Macon, Ga. This firm publishes the periodical entitled *Dental Luminary*, many of the articles which have appeared in its columns being from the pen of Dr. John P Holmes. The dental profession of Georgia, and also adjoining States will sadly miss Dr. J. P. Holmes, as he manifested great interest in all that related to dentistry, and was one of the most prominent members of his State Dental Society.

